# Calciphylaxis Cutis Associated With Fibroblast Growth Factor Receptor (FGFR) Inhibitor Therapy: A New Challenge

**DOI:** 10.7759/cureus.21478

**Published:** 2022-01-21

**Authors:** Paige Griffith, Jaroslaw Jedrych, Joel Sunshine, Daniel A Laheru, Mark Yarchoan

**Affiliations:** 1 The Sidney Kimmel Comprehensive Cancer Center, The Johns Hopkins University School of Medicine, Baltimore, USA; 2 Department of Dermatology, The Johns Hopkins University School of Medicine, Baltimore, USA

**Keywords:** hyperphosphatemia, fgfr2 inhibitor, pemigatinib, calciphylaxis, fgfr, fgfr2

## Abstract

Specific fibroblast growth factor receptor (FGFR) inhibitors have been developed to treat malignancies harboring fusions or rearrangements in FGFR2 or FGFR3. Here, we report a case of calciphylaxis cutis in association with FGFR inhibitor therapy in a patient with FGFR2 rearranged cholangiocarcinoma. Although calciphylaxis cutis typically arises in the setting of hyperphosphatemia and end-stage renal disease, this patient had preserved renal function, normal serum calcium, and only modestly elevated serum phosphorus levels, which is similar to other recent reports of calciphylaxis in patients receiving FGFR inhibitor therapy. Calciphylaxis cutis is a possible adverse event observed with FGFR inhibitor therapy, and the mechanism of calciphylaxis cutis in association with FGFR inhibitor therapy warrants further investigation.

## Introduction

Selective fibroblast growth factor receptor (FGFR) inhibitors were recently approved for the treatment of cholangiocarcinoma and urothelial carcinoma harboring fusions or rearrangements in FGFR2 or FGFR3. These therapies are associated with a variety of dermatologic adverse events including alopecia, nail toxicity, dry skin, and palmar-plantar erythrodysesthesia syndrome (PPES) [1]. Here, we report a case of non-uremic calciphylaxis cutis, a life-threatening syndrome of vascular calcification resulting in occlusion of microvessels in the subcutaneous tissue and painful necrosis of skin and subcutaneous tissues, in association with FGFR inhibitor therapy. The management of calciphylaxis cutis in a patient receiving FGFR inhibitor therapy is described.

## Case presentation

An 82-year-old female with advanced metastatic cholangiocarcinoma was initially treated with cytotoxic chemotherapy with stable disease as a best response lasting approximately six months. Molecular testing revealed the presence of a FGFR2 fusion, and she subsequently received pemigatinib at a dose of 13.5 mg oral daily, for 14 days on and seven days off. Her kidney function was within the normal range, and she had no other relevant past medical history.

**Figure 1:**
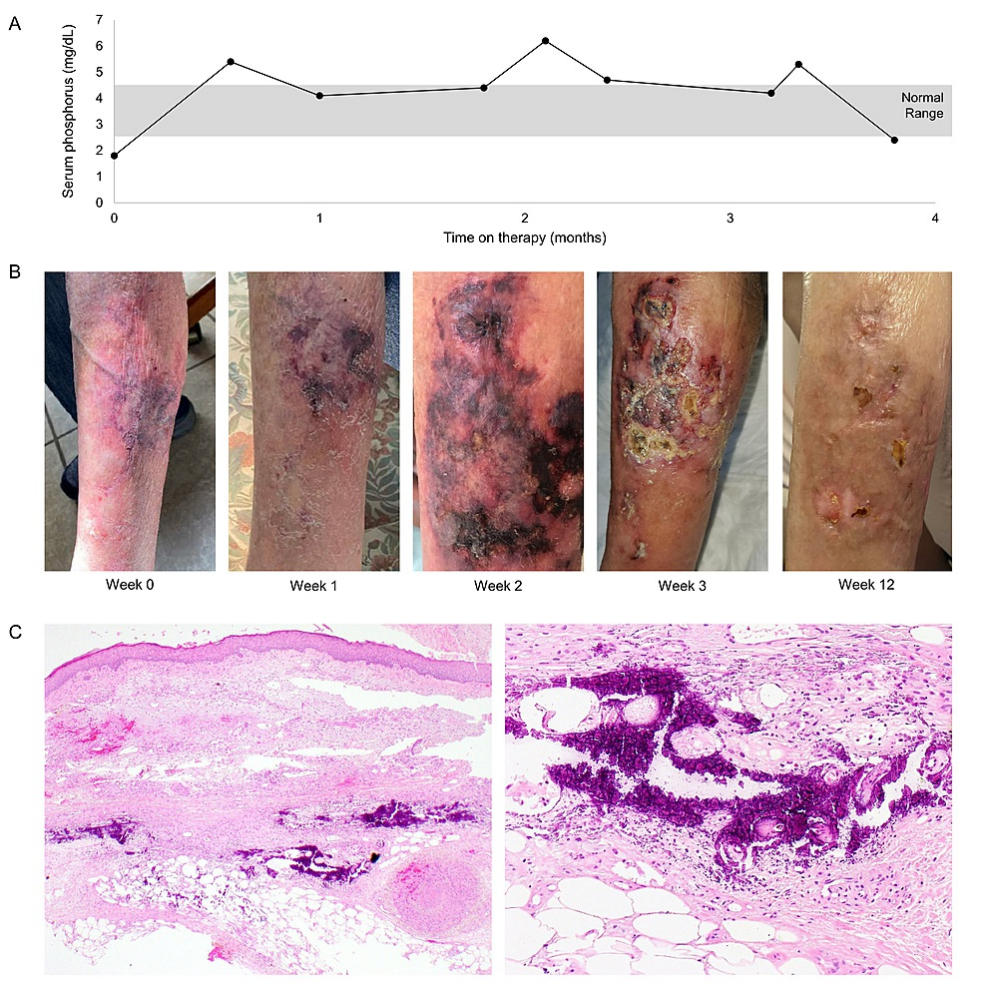
Calciphylaxis cutis in the setting of FGFR2 inhibitor therapy. (A) Serum phosphorus levels demonstrate only mild elevation over the course of pemigatinib therapy. (B) Progression of calciphylaxis cutis in the setting of FGFR2 inhibitor therapy and resolution at week 12 following drug cessation, wound care, antibiotics, and sodium thiosulfate infusions (STS). (C) Low power histological evaluation demonstrates diffuse subcutaneous necrosis, erythrocyte extravasation, and conspicuous calcifications within the dermis and subcutis (H&E, 20×). Higher magnifications display the interstitial and vascular distribution of microcalcifications. In areas, a pseudoxanthoma elasticum-like encrustation of the elastic fibers is noted (H&E, 200×).

She initially tolerated therapy well, with only minor fatigue and diarrhea attributable to her therapy. Her serum phosphorous level was checked regularly and fluctuated from as low as 2.2 to as high as 6.2 (normal range 2.7-4.5 mg/dL), but was generally in the range of 4-5 mg/dL (Figure 1A) during the treatment period. She was counseled on a low phosphate diet but was not started on a phosphorous binder. Her corrected serum calcium level was in the normal range. An initial restaging scan approximately eight weeks after starting pemigatinib demonstrated a partial response.

Approximately three months after starting pemigatinib therapy, she began to experience severe bilateral lower extremity pain. Physical examination of her lower extremities was initially unrevealing, but she subsequently developed progressive retiform purpura, eventually progressing to necrotic ulceration (Figure 1B). Her pemigatinib was discontinued. Lower extremity dopplers were obtained and demonstrated normal vascular flow. As the lesions progressed, a punch biopsy of the ulcerative lesion revealed perivascular erythrocytic extravasation and diffuse interstitial calcium deposition (Figure 1C). No specific immune deposits were seen using conjugates specific for IgG, IgA, IgM, C3, and fibrin. These pathologic findings were consistent with calciphylaxis cutis. She received oral antibiotics, triweekly wound care, and sodium thiosulfate (STS) infusions three times weekly but discontinued therapy after four weeks due to nausea associated with the STS therapy. Her calciphylaxis cutis improved over the course of three months. The patient provided informed consent to publish this report.

## Discussion

Specific FGFR inhibitors have established efficacy in multiple malignancies harboring FGFR2 or FGFR3 fusions or rearrangements. These include pemigatinib and infigratinib for cholangiocarcinoma with FGFR2 fusions or rearrangements [2,3], and erdafitinib for urothelial carcinoma with FGFR3 fusions or rearrangements [4]. These agents have variable selectivity for FGFR1-4 as well as the vascular endothelial growth factor receptor (VEGFR2). All of these agents are associated with on-target and off-target toxicities, including elevated phosphorus, alopecia, palmar-plantar erythrodysesthesia syndrome (PPES), stomatitis/mucositis, and nail changes.

Non-uremic calciphylaxis cutis is a very rare, potentially adverse, event with only a few cases reported in association with FGFR inhibitor therapy to date [5-7]. Calciphylaxis is characterized by calcium deposition into the microvessels, leading to vascular compromise and necrosis of subcutaneous tissues [8]. Although usually identified in the setting of hyperphosphatemia and end-stage renal disease (ESRD), our patient had preserved renal function, similar to other cases of FGFR inhibitor-associated calciphylaxis [5-7].

The mechanism of calciphylaxis in association with FGFR inhibitor therapy remains unclear. Hyperphosphatemia is a common side effect of FGFR inhibitors, and it has been proposed previously that an elevated serum calcium-phosphate product may mediate the link between FGFR inhibitors and calciphylaxis [1]. However, phosphorus levels were only modestly elevated in the case presented here. FGFR inhibitors may cause a compensatory elevation in FGF-23, a circulating hormone involved in phosphate regulation that is proposed to promote vascular calcification and is a risk factor for mortality in end-stage renal disease, independent of phosphate concentration [9]. FGFR inhibitors may alternatively promote vascular calcification through direct effects on FGFR signaling in target tissues [1]. The mechanism of calciphylaxis cutis in association with FGFR inhibitor therapy warrants further investigation.

## Conclusions

Calciphylaxis cutis is a possible adverse event observed with FGFR inhibitor therapy. Although considered a rare disease in the general population, clinicians treating with FGFR inhibitors should be aware of this possible adverse event as early identification and intervention may improve patient outcomes. The precise mechanism of calciphylaxis cutis in association with FGFR inhibitor therapy is unclear and warrants further investigation.
